# What Is a Major Operation?

**Published:** 1913-01-04

**Authors:** 


The Hospital

The Workers' Newspaper of Administrative Medicine and Institutional Life, Administration, National Insurance and Health.

No. 1383, Vol. LIII. SATURDAY, JANUARY 4, 1913. PRICE ONE PENNY.

### WHAT IS A MAJOR OPERATION?

Considerable discussion has been waged recently in American medical societies concerning the immediate and remote after-effects of tonsillectomy. It is well known that total excision of the tonsils in preference to partial excision, enucleation or tonsillectomy in preference to tonsillotomy and the guillotine operation, is regarded as the operation of choice. Those who habitually practise this method state that it is safe, simple, and satisfactory. The supporters of the older method, however, allege that complete enucleation is not, like the guillotine method, a minor operation, but is a grave business to which the indispensable preliminaries are a thorough examination of the patient's blood and the most careful avoidance of anything likely to promote sepsis or shock. In other words, tonsillectomy, they say, is a major operation, preferably to be performed under full general anæsthesia, with an aseptic technique that should correspond to that employed in an abdominal operation. Those who hold this view bring forward a list of fatalities and catastrophes that at first sight seems convincingly in favour of their conclusions. Deaths have been recorded after tonsillectomy from hæmorrhage, from shock, from sepsis inducing purulent endocarditis and infarction, from embolism, and from tetanus. Grave after-effects, such as contraction through scarring, phlegmonous inflammation of the mouth and throat, and pharyngeal ulceration, have been reported. But it is well to bear in mind that all these catastrophes have also followed the less severe operation of tonsillotomy, and that they may, indeed, follow any operation, however much the modern surgeon may regard it as minor in the sense that it needs no elaborate armamentarium and no staff of skilled assistants. There is no exact line of demarcation between minor and major operations. So many conditions have to be taken into consideration as influencing factors that the surgeon cannot possibly, without laying himself open to the charge of culpably hazarding failure, claim that one particular operation is in all circumstances a minor one. The mere extraction of a tooth from the jaw of a patient whose blood coagulation is imperfect is as "major" an operation in the sense that it entails immediate and remote dangers and calls forth the exercise of the greatest skill and presence of mind on the part of the operator, as an amputation of the leg in the same patient. In a hæmophilic patient, as a matter of fact, most surgeons would prefer to do an amputation to doing an oral operation, since in the former the surgeon's power of controlling the hæmorrhage is infinitely surer. Sir James Paget, in that classical article which every practitioner should read and digest, laid stress on the fact that the simplest and easiest operations demand from the operator the same careful consideration of possible eventualities and the same methodical preparation in cleanliness and technique as do the so-called major ones. Who is likely to forget the instances this great teacher has cited from his own practice, particularly the case of the diabetic patient on whom an operation of convenience for the removal of a small sebaceous cyst was performed with disastrous results? Every surgeon and every practitioner can cull from his own case-book reminders of the same lesson. Attention to detail is the practice of perfection. As in chess, the smallest move may mean the loss of the game, so in a surgical operation the neglect of a necessary preliminary precaution may land patient and surgeon in the greatest difficulties. While we are accustomed to regard as major operations those in which manipulative skill and inherent technical difficulties are predominant, such a classification is merely one of convenience. Nor is it possible to take the length of time and the duration of an operation as criteria. Tonsillectomy, for example, in simple cases is an operation of a few minutes; all the armamentarium necessary consists of a forceps to pull the gland forwards and inwards, and a dissector or blunt scissors to divide the circumvallating mucosa. It is an operation that can be, and generally is, successfully done under local anæsthesia; that may be carried out by an operator who has comparatively little dexterity and special skill, and that is usually followed by no worse results than the guillotine operation. But the very fact that this long list of dangers has been compiled makes it evident at once that tonsillectomy is not an operation to be lightly or carelessly done. When we say this we at once press home the fact that no operation, however easy or simple, should be decided upon and executed without care, prevision, forethought, and precaution. The crux of the whole question where minor surgery ends and major surgery starts lies in this, that the terms surgeons employ are misleading to the uninitiated public. To the layman there should be, and must be, no such thing as a minor operation. He should be taught that no surgical operation that involves anæsthesia, whether such anæsthesia be local or general, is wholly devoid of danger. It has been held in a German court of law that a surgeon ought to warn his patient of all the possible complications and consequences of operation. While we do not think it wise to urge that an omission in this respect should penalise the surgeon, we think that every operator, in his own interests, must deem it prudent to lay before the patient the possibilities of failure in every operation undertaken for the relief of inconvenience or physical danger. To hold that only a so-called major operation demands such precautionary measures is to discount the whole chapter of calamities in surgery which Pirogoff and Paget contributed to the literature, and to court dangers which may easily be guarded against. Tonsillectomy is no exception to the rule. It is undoubtedly a more thorough and more radical method of eliminating the septic focus in the pharynx than the older guillotine method, and it deserves wider popularity than it has yet attained. For that very reason it is necessary to take care that it be not brought into disrepute by careless and negligent operators whose misfortunes can serve only one useful purpose—namely, to point the moral that the profession ought by this time thoroughly to understand.

